# Efficient photosynthesis of carbon monoxide from CO_2_ using perovskite photovoltaics

**DOI:** 10.1038/ncomms8326

**Published:** 2015-06-11

**Authors:** Marcel Schreier, Laura Curvat, Fabrizio Giordano, Ludmilla Steier, Antonio Abate, Shaik M. Zakeeruddin, Jingshan Luo, Matthew T. Mayer, Michael Grätzel

**Affiliations:** 1 Laboratory of Photonics and Interfaces, Institute of Chemical Sciences and Engineering, School of Basic Sciences, Ecole Polytechnique Fédérale de Lausanne (EPFL), Station 6, CH-1015 Lausanne, Switzerland

## Abstract

Artificial photosynthesis, mimicking nature in its efforts to store solar energy, has received considerable attention from the research community. Most of these attempts target the production of H_2_ as a fuel and our group recently demonstrated solar-to-hydrogen conversion at 12.3% efficiency. Here, in an effort to take this approach closer to real photosynthesis, which is based on the conversion of CO_2_, we demonstrate the efficient reduction of CO_2_ to carbon monoxide driven solely by simulated sunlight using water as the electron source. Employing series-connected perovskite photovoltaics and high-performance catalyst electrodes, we reach a solar-to-CO efficiency exceeding 6.5%, which represents a new benchmark in sunlight-driven CO_2_ conversion. Considering hydrogen as a secondary product, an efficiency exceeding 7% is observed. Furthermore, this study represents one of the first demonstrations of extended, stable operation of perovskite photovoltaics, whose large open-circuit voltage is shown to be particularly suited for this process.

## Abstract (web summary)

Artificial photosynthesis is a means of harnessing solar energy to generate fuels but has traditionally been exploited for the generation of hydrogen. Here, Schreier *et al*. instead employ a perovskite photovoltaic device to effect the solar conversion of CO_2_ to CO with high efficiency.

## Graphical abstract

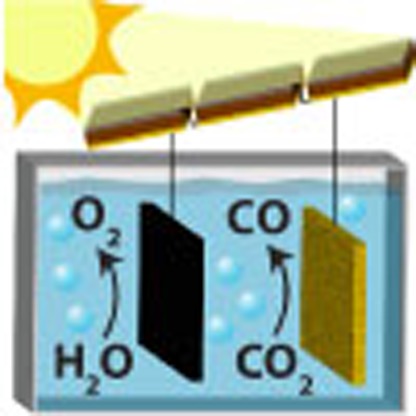

Using sunlight towards the generation of value-added chemicals entails capturing its energy in chemical bonds, a form of energy that can be readily stored and transported, thereby solving these two key challenges of solar light exploitation. This approach, called ‘artificial photosynthesis'^1^, has been mainly directed towards sunlight-driven splitting of water to generate H_2_ and O_2_, and solar-to-hydrogen energy-conversion efficiencies exceeding 10% have been demonstrated^2^,^3^,^4^. Although H_2_ is an important fuel and chemical feedstock, a desirable alternative is to target the direct production of carbon-based fuels, which allows for better integration into the existing energy infrastructure. These fuels can be accessed by the electrochemical reduction of carbon dioxide (CO_2_), in a process that comes a step closer to natural photosynthesis and simultaneously holds the promise of closing the anthropogenic carbon cycle^5^,^6^. Compared with water splitting, electrochemical CO_2_ reduction presents considerably greater challenges, including product selectivity, electrolyte constraints and large voltage requirements. The combination of these effects makes sunlight-driven CO_2_ reduction difficult to achieve a desirable product with high efficiency^7^.

The emergence of metal halide perovskites has attracted great attention in the photovoltaic community due to a combination of advantages, such as low-cost synthesis, abundant materials, and rapidly climbing efficiencies, presently certified at 20.1%, competing with the commercially available silicon solar cells^8^,^9^,^10^. Our group was recently able to demonstrate a simple system, achieving a striking 12.3% solar-to-hydrogen efficiency by the use of perovskite photovoltaics, thereby showing a promising path towards the realization of high-efficiency solar-to-fuel conversion devices^3^.

Here, extending our previous work in an attempt to get closer to carbon-based photosynthesis and taking advantage of the high open-circuit voltage of perovskite photovoltaics, we demonstrate the efficient reduction of CO_2_ to carbon monoxide (CO) driven solely by simulated sunlight using water as the electron source. Using series-connected perovskite photovoltaics and high-performance catalyst electrodes, we achieve a solar-to-CO efficiency exceeding 6.5%, setting a new benchmark in sunlight-driven CO_2_ conversion. Considering also hydrogen, which is generated as a secondary product, a total solar-to-fuel efficiency exceeding 7% is achieved. It has to be noted that in addition to the aforementioned results, this study represents one of the first demonstrations of extended, stable operation of perovskite photovoltaics under a real load.

## Results

### Carbon monoxide as a photosynthetic target

Among all the products obtained by the reduction of CO_2_ (ref. 11), CO stores the largest amount of energy per molecule and is an important bulk chemical in manufacturing^12^. Importantly, through the established gas to methanol and Fischer–Tropsch processes, as well as novel electrochemical methods, CO can be converted into a large number of carbon fuels and other commodity chemicals^13^,^14^,^15^. Recently, researchers have proposed a two-step process towards the solar-driven synthesis of hydrocarbon fuels using CO as an intermediate^16^,^17^,^18^. Therefore, CO is one of the most attractive targets of artificial photosynthesis.

A complete process for the light-driven synthesis of carbon monoxide must be based on a sustainable and balanced reaction, consuming only CO_2_. To match the reduction of CO_2_ at the cathode, an oxidation reaction must occur at the anode, which supplies the electrons consumed in the reduction and regenerates the protons used to accept oxygen from CO_2_. This is a seldom-considered requirement that plays an important role in overall device performance^19^. Many studies are presently concerned solely with the cathode process, employing sacrificial and therefore unsustainable reactions on the anode side. Here, an aqueous system is employed where water oxidation continuously provides a proton source to accept oxygen from CO_2_, leading to the reactions defined in Table 1.

**Figure 1:**
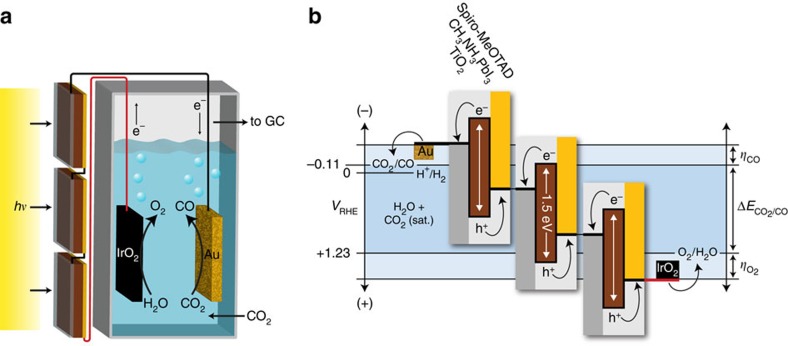
Sunlight-driven CO_2_ reduction device. (**a**) Schematic of the device combining photovoltaics with an electrochemical cell. (**b**) Generalized energy diagram for converting CO_2_ into CO with three perovskite solar cells. The series-connected photovoltaics produce a voltage sufficient to overcome the sum of the reaction free energy (*ΔE*) and the reaction overpotentials (*η*) at the electrodes.

The production of CO from CO_2_ requires 259 kJ mol^−1^ of free energy (22 kJ mol^−1^ greater than water electrolysis), corresponding to a voltage of 1.34 V. However, both the cathodic and anodic half reactions suffer significant kinetic overpotentials (*η*), which must be overcome to drive the reaction at meaningful rates. These overpotentials depend largely on the nature of the electrodes and on the constraint that electrochemical CO_2_ reduction must be performed at near-neutral pH, as discussed below. Gold (Au) is known to be the best catalyst in terms of overpotential for selective cathodic CO evolution^12^,^20^. However, a number of different products can be generated by reduction processes on Au, including several carbon products as well as hydrogen from aqueous proton reduction. This creates a potential-dependent product selectivity, which must be accounted for when defining the cathode operating potential. On the anode side, iridium oxide (IrO_2_) is known to be a top performer for the oxygen evolution reaction^21^, but suffers an overpotential increase in near-neutral conditions. As represented schematically in Fig. 1 and as described below, considering the thermodynamic voltage and the overpotentials for both electrodes, a voltage of at least 2 V is demanded to drive efficient and selective CO evolution from CO_2_.

### Preparation and characterization of catalyst electrodes

**Figure 2:**
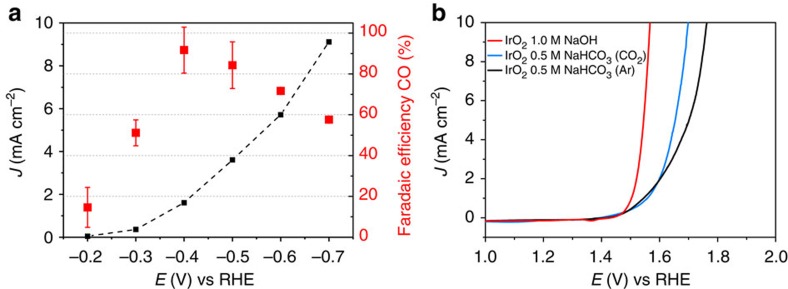
Characterization of CO_2_ reduction and H_2_O oxidation electrodes. (**a**) Electrochemical performance and FE towards CO production of oxidized Au electrodes in CO_2_-saturated aqueous solution of 0.5 M NaHCO_3_. Error bars correspond to the s.d. of repeated gas measurements. (**b**) Electrochemical performance of IrO_2_ towards water oxidation in solutions of 1.0 M NaOH and 0.5 M NaHCO_3_ under Ar and CO_2_ saturation.

To enhance the catalytic performance of Au, oxidized cathodes were prepared by electrochemical anodization as previously described^22^. After exposure to reaction conditions, these electrodes exhibit a highly porous structure of metallic Au with an increased surface area (Supplementary Fig. 1). The electrocatalytic performance of this material was assessed at various potentials in a three-electrode configuration in CO_2_-saturated 0.5 M NaHCO_3_ aqueous electrolyte (pH 7.2) and gas chromatography analysis was performed *in situ* to monitor the product. In Fig. 2a, the cathodic current density and Faradaic efficiency (FE) of CO production are reported as a function of electrode potential versus the reversible hydrogen electrode (RHE). Carbon monoxide production starts to be observed at 90 mV overpotential (−0.20 V versus RHE), confirming the impressive activity of gold towards this reaction. The CO selectivity peaks at around −0.4 V versus RHE, exceeding 90% FE towards CO, then decreases again at more negative potentials. The remaining balance of current primarily goes to the reduction of aqueous protons to generate hydrogen. Owing to this potential-dependent product selectivity, an effective overpotential for optimal CO yield is around 300 mV on this cathode.

As mentioned above, aqueous carbon dioxide reduction processes are constrained to near-neutral environments. This is because in acid most known CO_2_ reduction catalysts favour H_2_ evolution over reducing carbon dioxide, whereas in base dissolved CO_2_ spontaneously converts to carbonate, which is unreactive as a substrate in electrochemical CO evolution^12^. In a complete system, the anode and cathode must be operated in the same solution to avoid introducing chemical bias based on concentration gradients, masking the true operating voltage. Commonly, CO_2_ reduction is carried out in aqueous bicarbonate electrolytes, which, on saturation with CO_2_, results in a solution buffered at pH 7.2. This presents a challenge for the anodic reaction, as although many electro-catalysts have demonstrated high catalytic activity towards the oxygen evolution reaction^21^, they are typically most efficient in either strongly alkaline or acidic solutions. Oxygen evolution from near-neutral solutions has received less attention^23^,^24^, but nonetheless plays an important role in complete CO_2_ reduction cells. As we observed that materials based on nickel led to poisoning of the Au cathode, IrO_2_ was selected as anode material here because of its great stability and excellent performance towards the oxygen evolution reaction^21^. Current–voltage curves of IrO_2_ electrodes, prepared by thermal decomposition of H_2_IrCl_6_ on Ti foil, are shown in Fig. 2b. In alkaline solution (1 M NaOH), these electrodes show comparable performance to what has been reported previously^25^. In 0.5 M NaHCO_3_, the onset is shifted to slightly more positive values and it was found that the catalyst showed better performance in a CO_2_-saturated electrolyte than under Ar. This effect might be attributed to the increased buffer strength of carbonate in the presence of CO_2_. Under CO_2_ photolysis conditions, the anode reaches 5 mA cm^−2^ at an overpotential of 400 mV.

### Integrated device characterization

**Figure 3:**
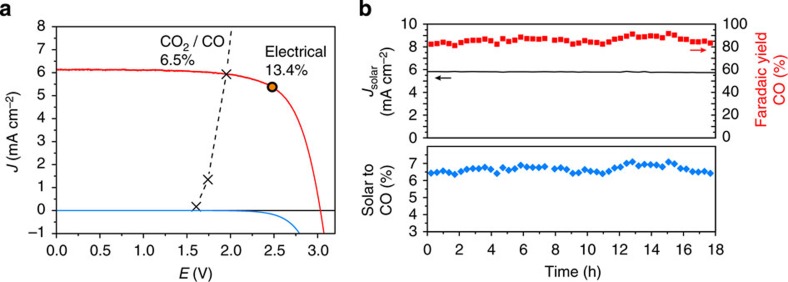
Characterization of the complete device. (**a**) *J*–*V* curves of three series-connected perovskite cells under simulated AM 1.5G 1 Sun solar irradiation and in the dark, overlaid with the matched *J*–*V* characteristic of the CO_2_-reduction and oxygen-evolution electrodes. The maximum power point of the photovoltaics is designated with a dot. (**b**) Current density, CO yield and solar-to-CO conversion efficiency of the device during an 18-h stability test.

Considering the performance of both electrodes, an overall overpotential of about 700 mV is expected for selective production of CO, therefore necessitating a driving force of 2 V or more. This is visualized in their current–voltage response in a two-electrode configuration (Fig. 3a), where additional ohmic losses are taken into account (see Methods). Driving this reaction with sunlight requires a device producing voltages considerably higher than conventional photovoltaics (PVs). In this study, three perovskite cells connected in series were employed (see Methods), producing an open-circuit voltage of 3.1 V and a short-circuit current density of 6.15 mA cm^−2^ (Fig. 3a, red). The operating point of the complete device can be predicted by the intersection of the electrode and photovoltaic curves^3^,^26^, which, in this case, falls on the outer end of the plateau current of the photovoltaic—not far from the maximum power point for solar-to-electric energy conversion. Intersecting in this relatively flat region of the perovskite *J*–*V* characteristic enables a stable system with respect to small perturbations such as fluctuations in the light intensity and performance fluctuations of the catalysts. The data predict a device current density of ∼5.93 mA cm^−2^ (normalized to the total illuminated area of the photovoltaics), which is close to the value observed on the complete assembled device.

For long-term testing of the complete device, a CO_2_-saturated solution of 0.5 M NaHCO_3_ was used as electrolyte and the PV cells were kept in a transparent chamber under a constant flow of argon. On exposure to simulated sunlight, the series-connected perovskite tandem cell produced an absolute current of 1.65 mA (for a total illuminated area 0.285 cm^2^). To establish the cathode-operating potential at −0.4 V versus RHE, at which CO yield on Au is maximal, the cathode area was adjusted to 1.0 cm^2^ to achieve the desired current density as defined by the catalyst behaviour shown in Fig. 2a. After initial equilibration of both the catalyst and the PV cells, the system was allowed to run without any external bias under constant illumination for more than 18 h (Fig. 3b). The current density remained constant at about 5.8 mA cm^−2^ and the minimal change in observed photocurrent verifies the excellent stability of not only the anode and cathode, but also of the three perovskite photovoltaics that are transferring power to the catalyst load. To the best of our knowledge, this is the first study of extended testing of CH_3_NH_3_PbI_3_ perovskite photovoltaics under actual load conditions, confirming that stable operation of these devices can be achieved.

Evolved gases were periodically analysed by gas chromatography and the CO signal was related to the measured currents to determine the FE of sunlight-driven CO_2_ reduction to CO. As shown in Fig. 3b, during the 18-h period of operation, the FE varies between 80% and 90%. The energy-conversion efficiency for a given species (solar-to-CO in this example) is defined by equation (1):


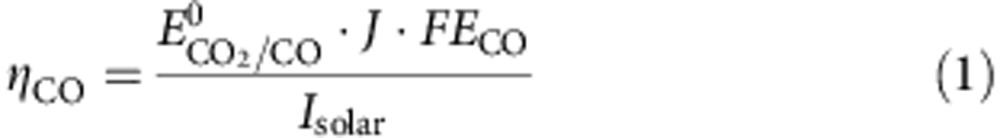


where 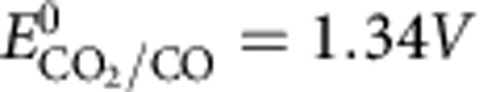 is the thermodynamic energy stored in the CO_2_/CO couple, *J* is the observed current density, *FE*_CO_ is the faradaic efficiency towards CO formation and *I*_solar_ is the solar power density. The measured current density and FE combine to yield a CO_2_ reduction efficiency >6.5%, a new benchmark exceeding greatly the efficiencies previously reported on systems driven by Si photovoltaics^27^,^28^. In addition, considering hydrogen that is formed as a secondary product at an average faradaic yield of 10.5% over the whole experiment, a solar-to-fuel efficiency exceeding 7% was achieved using this system. Although our study focuses on gas-phase products, we note that anodized gold catalysts have been reported to also produce liquid-phase products, albeit in very small amounts^22^.

To understand the behaviour of each component, the potentials of the anode, cathode and each photovoltaic cell were monitored over time, as plotted in Supplementary Fig. 2a. It can be seen that a distinct equilibrium takes place between the three series-connected photovoltaic cells, whereas the potentials at the anode and cathode remain fairly constant during the course of the experiment. *J*−*V* curves of the series-connected PV before and after testing are shown in Supplementary Fig. 2b. As the perovskite photovoltaics are tested over an extended amount of time and the efficiency is defined by the actual current density during operation (equation (1)), hysteretic behaviour does not affect the determination of the CO_2_ reduction efficiency^29^. In this configuration, the performance of the device is largely dictated by the interplay between the Au cathode and photovoltaic current–voltage behavior, as the anode shows a very steep *J*–*V* behaviour compared with the other system components and thus only negligibly influences the operating voltage.

## Discussion

In this work, we demonstrated a highly efficient and unassisted photolytic system for the reduction of carbon dioxide to carbon monoxide using water as electron source, reaching benchmark solar-to-CO efficiencies over 6.5%. This system, driven by three perovskite photovoltaics, was shown to be stable over 18 h. Remarkably, the extended operation under load demonstrated the ability of the photovoltaics to maintain the necessary voltage for selective photolytic synthesis of CO from CO_2_ in the long term, encouraging further work on perovskite stability. Their large open-circuit voltage constitutes a major strength of perovskite photovoltaics. As a consequence of this, three of these in series-connected cells were more than sufficient for CO production to be driven with high efficiency, whereas conventional photovoltaics such as Si, on the other hand, require at least four cells to achieve voltages sufficient to drive water splitting and CO_2_ reduction efficiently^4^.

Despite the remarkable efficiencies that were observed here, there is still room for improvement. The use of more abundant electrode materials will benefit the applicability of this catalytic approach to industrial implementation. For instance, transition metal or mixed metal cathodes have shown promise towards CO evolution, although their performances are still less desirable than for gold^30^. In addition, alternative anode materials for the oxygen evolution reaction in near-neutral pH, such as Co-Pi and Ni-Bi^23^,^24^, should be examined, keeping in mind that metal contaminants in solution present the risk of poisoning the activity of the CO_2_-reducing cathode^12^. As this photovoltaic system produces a slight excess photovoltage, this design could accommodate the use of higher-overpotential but cheaper electrode materials and provide great potential for the selective synthesis of other products.

Side reactions due to cross-over of dissolved products (for instance, re-oxidation of CO at the anode) constitute a loss channel in this single-compartment system (Supplementary Fig. 3). Further efficiency improvements can be expected on incorporating a separator to prevent product diffusion between electrode compartments. The use of a membrane can, however, impart a significant overpotential because of the buildup of a pH gradient, as has been previously reported^31^,^32^. Such a gradient and overpotential buildup would be detrimental to the operation of a CO_2_ reduction cathode, which exhibits a pH- and potential-dependent product distribution. The development of an effective membrane for neutral electrolysis could enable even higher efficiencies in such a system and may represent a promising avenue for continued research. Nevertheless, the solar-to-CO efficiency reported here is unaffected from cross-over, as it is derived from real-time measurements of the evolved gases.

The concept presented here shows that record performance can be reached through simple device design. It therefore carries a strong encouragement towards simplifying systems for solar fuel generation and opens up a new path toward the efficient storage of solar energy in carbon based fuels, which has the potential to solve numerous challenges currently faced by the field of renewable energy utilization.

## Methods

### Electrode preparation and characterization

Oxidized gold cathodes were prepared with a modified method as described in ref. 22. Gold foil (99.97%, Chempur, Germany) was cleaned in aqua regia mixed with deionized water (18.2 MΩ) and subsequently oxidized in 0.5 M H_2_SO_4_ (96%, ‘extra pure', Acros Organics) by applying square pulses between 1.183 and 3.183 V versus Ag/AgCl (KCl sat.) at 500 Hz using a potentiostat (Interface 1000, Gamry USA). IrO_2_ anodes were prepared as follows: titanium foil (99.7%, 0.25 mm, Sigma Aldrich) was etched for 60 min in boiling 1 M oxalic acid (⩾97%, anhydrous, Fluka). Subsequently, 30 μl of 0.2 M H_2_IrCl_6_ (99.9%, hydrate, ABCR) in isopropanol (ACS Reagent, Merck) were drop cast on the foil. This was followed by drying at 70 °C for 10 min and calcination at 500 °C for 10 min in air. The step was repeated three times on each side of the Ti foil. IrO_2_ (6.3 mg) was deposited on each side. Both electrodes were characterized before and after photolysis experiments by scanning electron microscopy (SEM; Supplementary Fig. 1) and X-ray diffraction (Supplementary Figs 4, 5). SEM micrographs were recorded using a Zeiss Merlin high-resolution SEM. X-ray diffraction measurements were performed on a Bruker D8 Discover X-ray diffractometer in the Bragg-Brentano Geometry using a CuK_α_ source (1.540598 Å) and a Ni β filter. A linear silicon strip detector was used (Lynx Eye). Scans were acquired from 2*θ*=15°–110° with a step width of 0.03° and scan rates between 2 and 10 s per step. The diffraction patterns were matched to the PDF-4+ database (ICDD) and to literature reports^33^.

### Photovoltaic cell preparation

Perovskite photovoltaic cells were prepared using a similar procedure as described in the literature^34^. Different from the previous method, the perovskite precursor solution was prepared by dissolving 1.1 M of lead iodide and methyl ammonium iodide in dimethylsulfoxide. The perovskite film was deposited by spin coating, using chlorobenzyl as antisolvent to control the crystallization. 2,2′,7,7′-tetrakis-(*N*,*N*-di-*p*-methoxyphenylamine)-9,9′-spirobifluorene (*spiro*-OMeTAD) was used as hole-transporting layer and it was doped with bis(trifluoromethylsulfonyl)imide lithium salt and tris(2-(1H-pyrazol-1-yl)-4-tert-butylpyridine)- cobalt(III) tris(bis(trifluoromethylsulfonyl)imide)^35^,^36^.

### Electrochemical device testing

Electrocatalytic testing was performed in CO_2_-saturated 0.5 M NaHCO_3_ (99.7%, Sigma Aldrich) at pH 7.2. Anode J-V curves were recorded in 1 M NaOH (Reactolab, Switzerland) and in 0.5 M NaHCO_3_ under Ar (Carbagas, Switzerland) and CO_2_ (Carbagas Switzerland) using a potentiostat (Interface 1000, Gamry USA). A Luggin capillary was used to minimize the iR-drop between the working and reference electrodes, therefore eliminating the need for applying iR correction, which is a common source of performance overestimation. The gold cathode performance was assessed by testing at different potentials in CO_2_-saturated 0.5 M NaHCO_3_ aqueous electrolyte (pH 7.2), each point for 2 h and the product was monitored at the same time by gas chromatography. These tests were performed in a 25-ml three-neck flask with an Ag/AgCl (KCl sat.) reference electrode (Metrohm, Switzerland) and sealed by septa (Suba Seal, Sigma Aldrich). A flamed Pt wire was used as counter electrode. CO_2_ was sparged into the electrolyte at 20.00 ml min^−1^ using a mass flow controller (Bronkhorst EL-Flow, The Netherlands) and the product gas was analysed in a gas chromatography apparatus (Trace ULTRA, Thermo USA) equipped with a ShinCarbon Column (Restek, USA) and a PDD detector (Vici, USA), which was calibrated with respect to certified gas standards (Carbagas, Switzerland). Combined *J*–*V* characteristic of the anode and cathode were derived from the data obtained on both electrodes, considering the series resistance of the cell and 4.5 cm^2^ as the surface area of the anode and adjusting the surface area of the cathode to match the PV current at 2 V at −0.4 V versus RHE as described below. During testing, perovskite cells were kept in a custom-made chamber, which was constantly flushed with argon gas (90 ml min^−1^). This chamber was illuminated using a 450 W Xe arc lamp (Lot Oriel) with a KG3 filter (Edmund Optics, USA). The total illuminated area of the perovskite cells was 0.285 cm^2^. The intensity of the light source was adjusted to match standard AM 1.5G sunlight at 100 mW cm^−2^ intensity. *J*–*V* curves of the cells were recorded from 3.1 to 0 V at a scan rate of 10 mV s^−1^. Electrocatalysis was performed as described above but with the Pt anode replaced by IrO_2_/Ti. Before the test, the cathode was activated at −0.4 V versus RHE until reaching a steady current. Similarly, the series-connected PV cells were activated at 2 V. No external bias was applied for the whole duration of the stability test. For the Au cathode to yield a maximum selectivity for CO, its surface area was adjusted to 1.0 cm^2^, which binds it to operate at −0.4 V versus RHE at the current supplied by the photovoltaics (as described above and seen in the catalyst *J*–*V* curve in Fig. 2a). The surface area of the anode was left constant at 4.5 cm^2^. The stable device current density of 5.8 mA cm^−2^ (normalized by illuminated area) corresponded to a net current of 1.65 mA and therefore electrode current densities of 1.65 and 0.37 mA cm^−2^ on the cathode and anode, respectively. The FE data were smoothed to correct for variations due to bubble formation and bubble breaking. Chopped illumination experiments were conducted to confirm the light dependence of current and electrode potentials (Supplementary Fig. 6). During testing, the current and voltage on each cell and on each electrode, as well as on the Ag/AgCl (KCl sat.) electrode were monitored using a Keithley 197 multimeter connected to an A/D converter (USB 6211, National Instruments, USA) and the data recorded using LabView (National Instruments, USA). The series resistance between the anode and cathode was evaluated by potentiostatic AC-impedance measurements between 1 MHz and 0.2 Hz at 2 V cell voltage and 10 mV perturbation (Supplementary Fig. 7), using the same potentiostat as above, and determined to be 22 Ω.

## Additional information

**How to cite this article:** Schreier, M. *et al*. Efficient photosynthesis of carbon monoxide from CO_2_ using perovskite photovoltaics. *Nat. Commun.* 6:7326 doi: 10.1038/ncomms8326 (2015).

## Supplementary Material

Supplementary Information Supplementary Figures 1-8 and Supplementary References

## Figures and Tables

**Table 1:** Redox processes involved in CO synthesis from CO_2_.

**Reaction**	**Standard electrode potentials (*****V*** versus RHE^*^)
CO_2_ (g)+2 H^+^+2 e^−^⇌ CO (g)+H_2_O (l)	–0.11
H_2_O⇌½ O_2_ (g)+2 H^+^+2 e^−^	+1.23
CO_2_ (g)⇌CO (g)+½ O_2_ (g)	*ΔE*=1.34 V

*RHE, reversible hydrogen electrode.
