# Supplementary material for: Efficient photosynthesis of carbon monoxide from CO2 using perovskite photovoltaics
Source: Nat Commun. 2015 Jun 11;6:7326. doi: 10.1038/ncomms8326 (PMC4699397; doi:10.1038/ncomms8326)
Supplement: Supplementary Information — Supplementary Figures 1-8 and Supplementary References [file ncomms8326-s1.pdf]

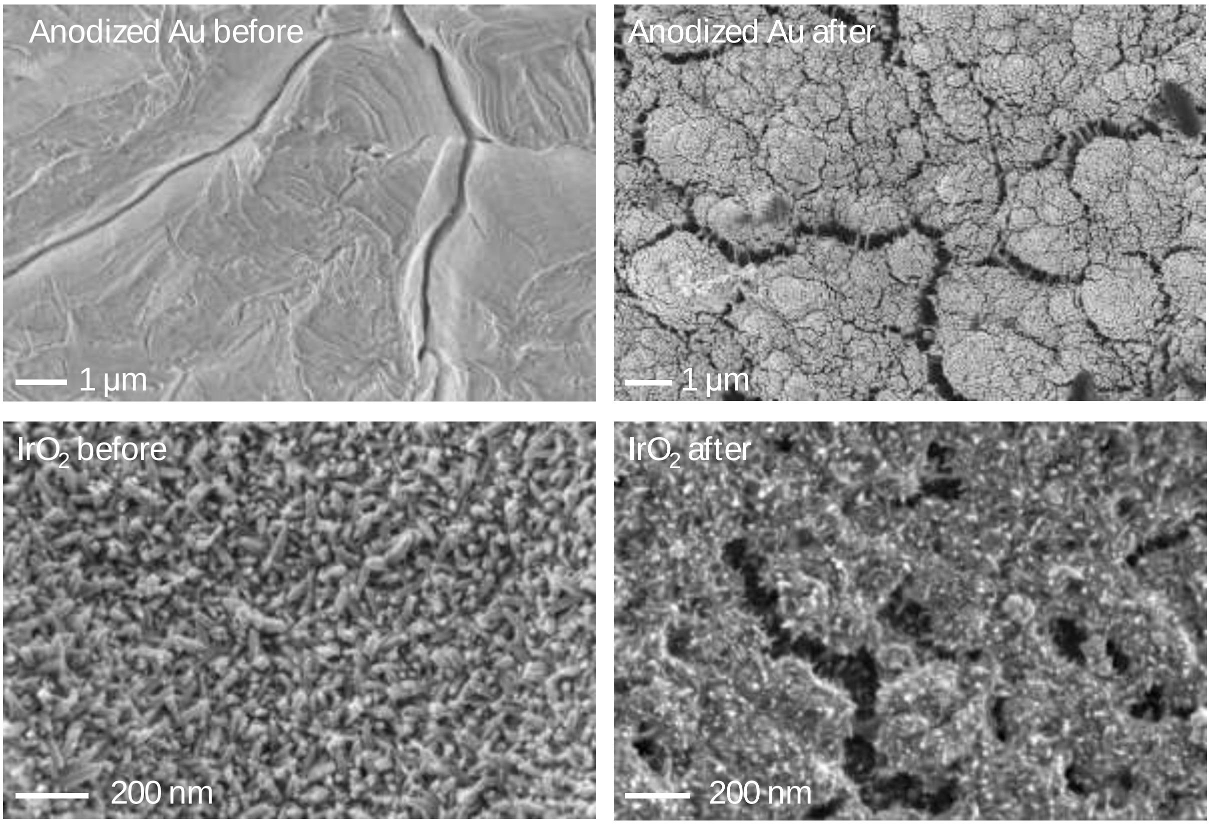

Supplementary Figure 1: SEM micrographs of anodized Au cathodes and IrO_2 anodes before and after CO_2 photolysis. During the photolysis experiment, the anodized Au cathodes develop a highly porous structure whereas the structure of the IrO_2 anodes stays relatively similar.

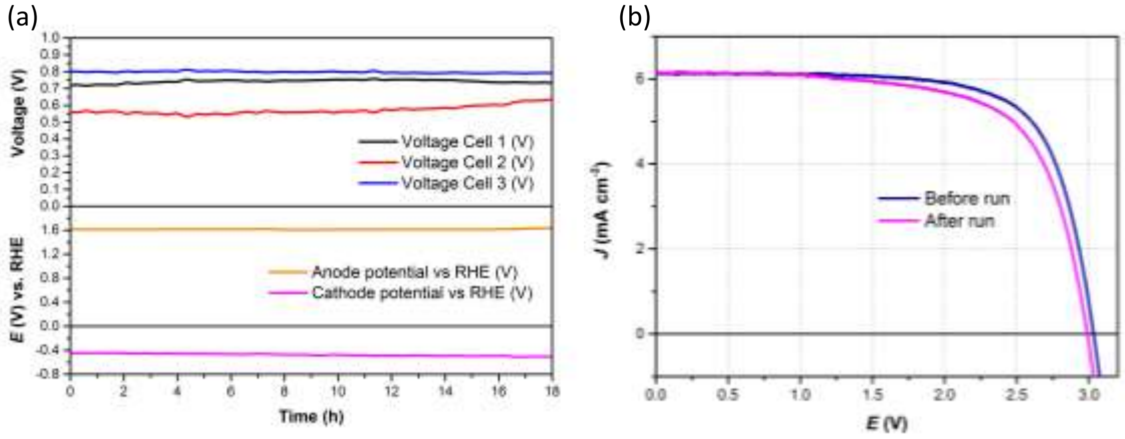

Supplementary Figure 2: (a) Evolution of each cell voltage and cathode and anode potential over the course of the experiment and (b) JV scans of the series-connected perovskite cells before- and after run. The perovskite solar cells behave in a distinct equilibrium to each other. The potentials of the electrodes stay fairly constant over time, however a slight increase in cathode potential can be observed.

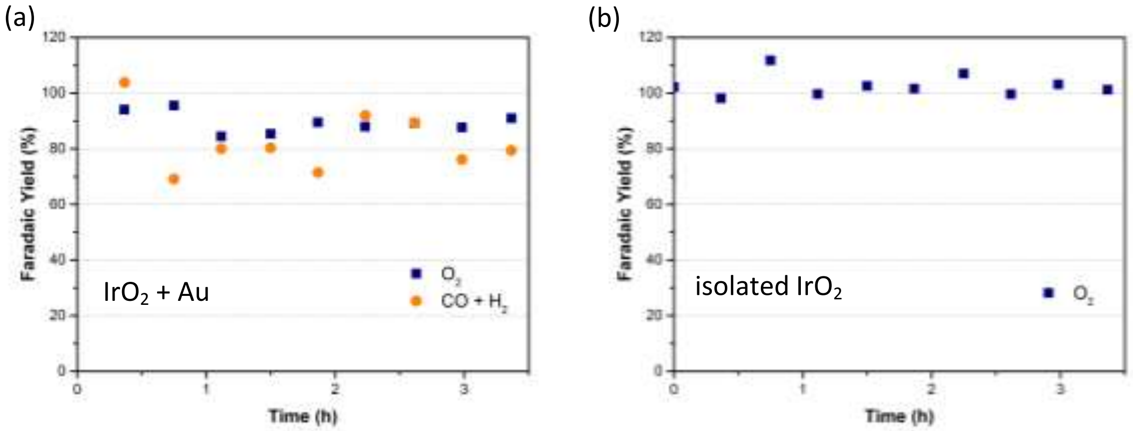

Supplementary Figure 3: Faradaic yields of anode and cathode products. In order to elucidate the efficiency of the anode process, in a separate experiment, in addition to CO and H_2, the concentration of O_2 was quantified in the outlet stream by gas chromatography. During this experiment, the cathode was polarized at -0.4 V vs. RHE while IrO_2 was used as anode in the same compartment. (a) The total yield of gaseous cathode products (CO + H_2) is comparable to the yield of O_2. The slightly lower amount of observed cathode products is attributed to the presence of liquid cathode products, previously reported to evolve from the cathode. [1] The total oxygen yield reached 90%, which is attributed to product crossover in the single-compartment system. (b) Conversely, in a control experiment in the same electrolyte, with the cathode separated from the anode by a ceramic diaphragm, the O_2 yield reached 100%, signalling that oxidation of cathode products, as well as reduction of anode products, constitute a possible loss channel and its elimination therefore an avenue for improving the solar-to-CO efficiency.

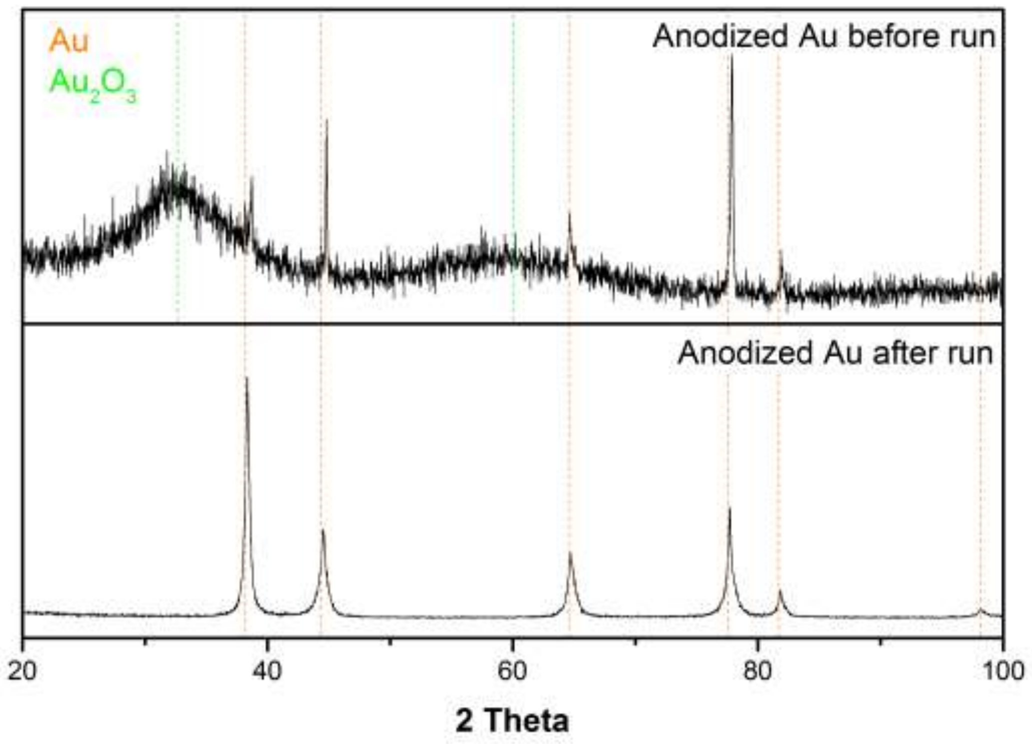

Supplementary Figure 4: XRD analysis of anodized Au cathodes before and after CO_2 photolysis. It can clearly be seen that anodization of gold has led to Au_2O_3 which is entirely reduced to metallic Au during the photolysis experiment. Au reference from JCPDS card #04-0784. Au_2O_3 reference from [2]. Weak lines were omitted for clarity.

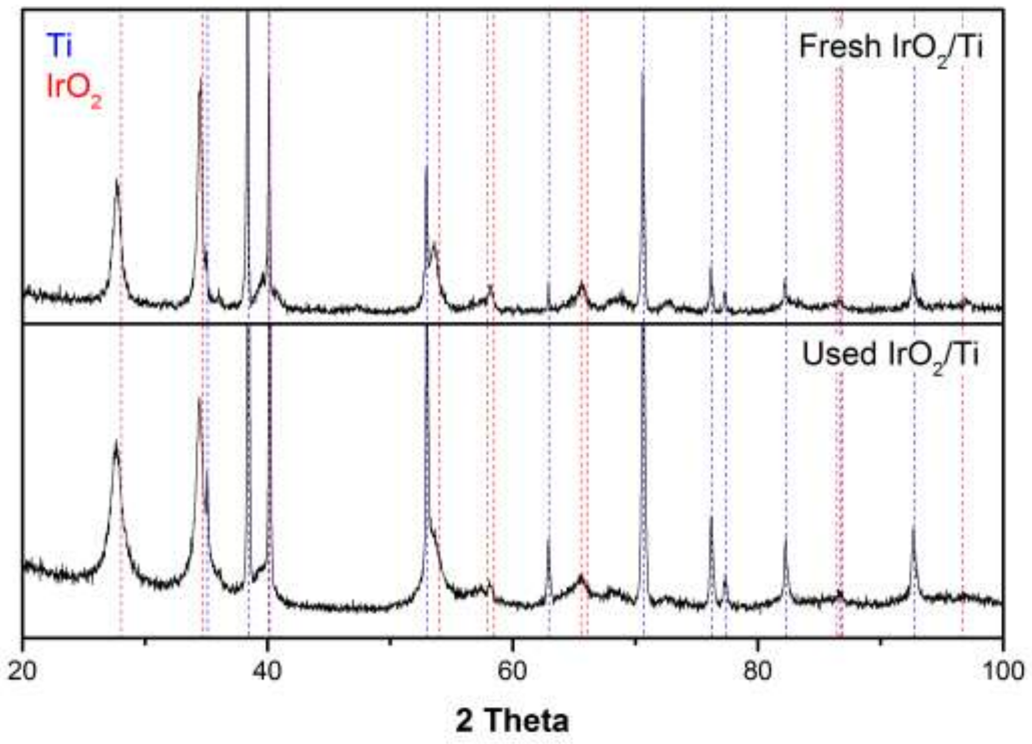

Supplementary Figure 5: XRD analysis of IrO_2 deposited on Ti foil before and after CO_2 photolysis. The presence of IrO_2 can be seen from the diffraction pattern. Only few differences are observed before and after testing, confirming the impressive stability of anodes based on IrO_2. Ti reference from JCPDS card #44-1294, IrO_2 reference from JCPDS card #15-0870. Weak lines were omitted for clarity.

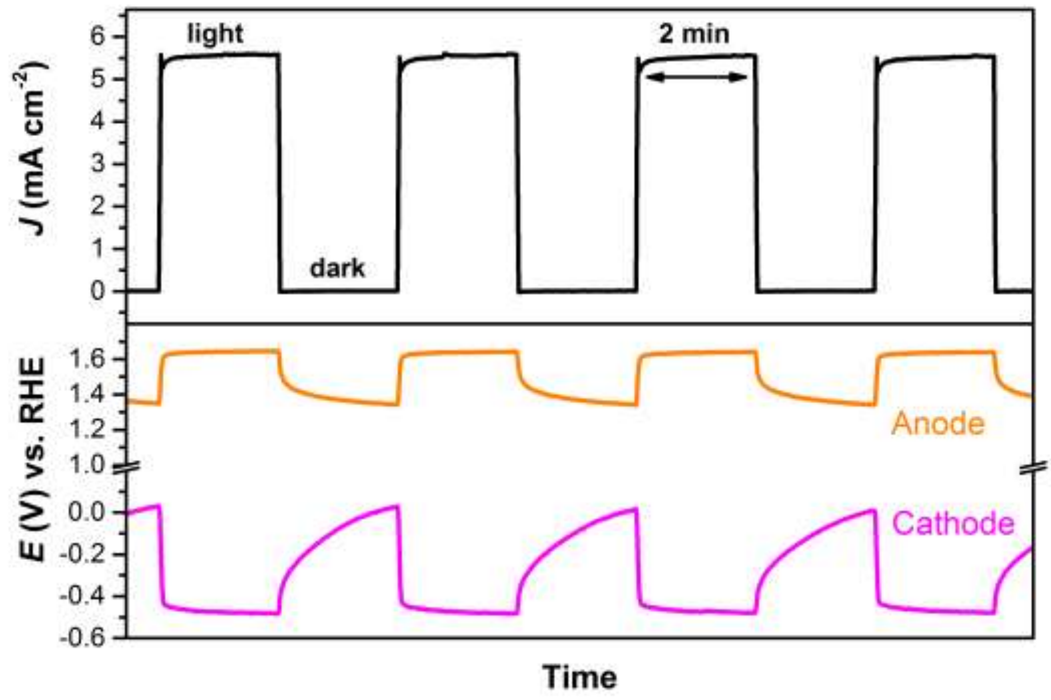

Supplementary Figure 6: Chopped illumination (2 minutes interval) with simultaneous measurement of the system current as well as cathode- and anode potentials vs. RHE. It can be seen that in the dark, no currents are observed. The equilibration of the system is slow, with the two minute interval not allowing for complete equilibration. The IrO_2 anode reaches its equilibrium much more quickly than the cathode.

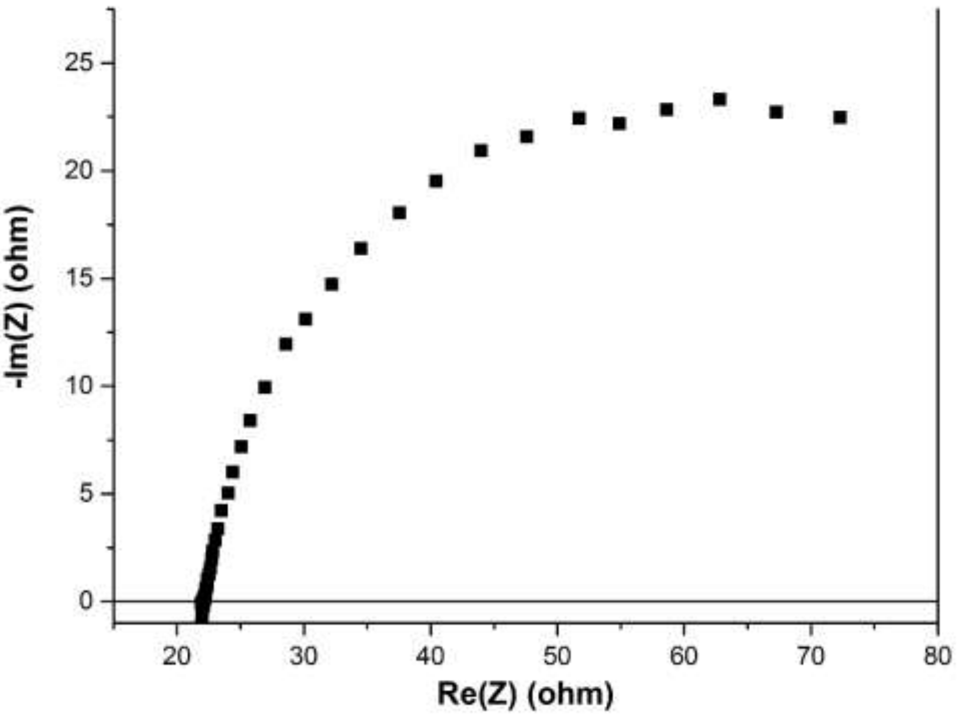

Supplementary Figure 7: 2-electrode AC-Impedance measurement for determination of the cell resistance. A 10 mV perturbation of a 2 V cell potential was applied, using a frequency range of 10^6 - 0.2 Hz. The series-resistance was determined to be 22 ohms.

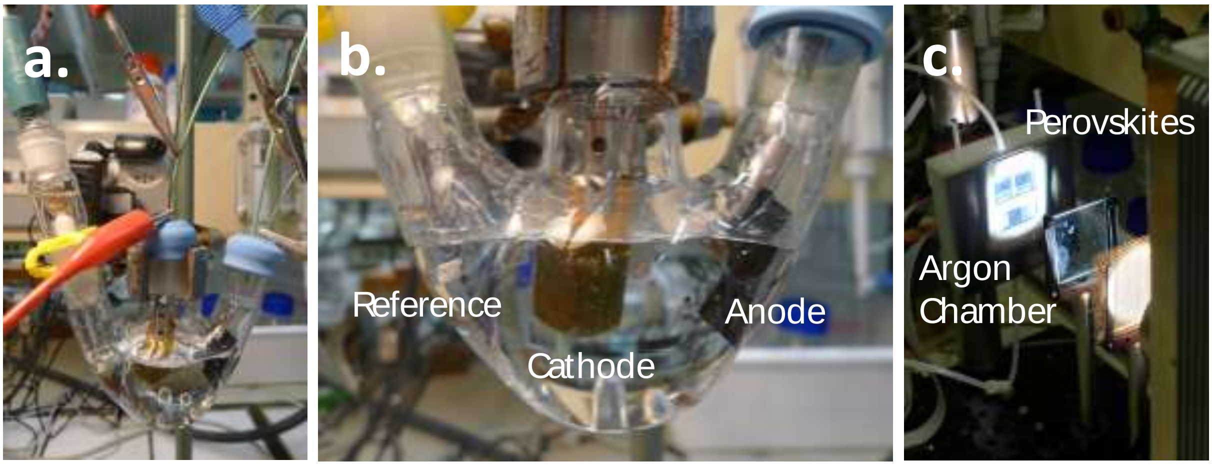

Supplementary Figure 8: Experimental setup. (a) A three-neck round-bottom flask was used for electrocatalysis. PEEK tubing was used to bring in CO_2 and remove gases for continuous analysis by gas chromatography. Various electrical contacts are used to connect the catalysts to the PV device and to log the potentials of all system constituents which are related to the potential of a Ag/AgCl (KCl sat.) reference electrode. (b) Anode, cathode and reference electrodes can be seen in the three-neck round bottom flask. Kapton® was used to mask the electrodes. (c) A gastight box was used to keep three perovskite cells under an argon atmosphere.

Supplementary References

1. Chen, Y., Li, C. W. & Kanan, M. W. Aqueous CO_2 Reduction at Very Low Overpotential on Oxide-Derived Au Nanoparticles. *J. Am. Chem. Soc.* **134,** 19969–19972 (2012).

2. Hore, S. et al. Carbonization of polyethylene on gold oxide. *J. Mater. Chem.* **18**, 5589–5591 (2008).
